# Tungsten Material Behavior under H_2_, D_2_, and He Plasma Interaction Conditions in the Framework of Fusion-Relevant Studies

**DOI:** 10.3390/ma16216853

**Published:** 2023-10-25

**Authors:** Cristian Stancu, Valentina Marascu, Anca Bonciu, Adrian Bercea, Silviu Daniel Stoica, Catalin Constantin

**Affiliations:** 1 Low Temperature Plasma Department, National Institute for Laser, Plasma and Radiation Physics, 409 Atomistilor Street, 077125 Magurele, Romania; cristian.stancu@inflpr.ro (C.S.); daniel.stoica@inflpr.ro (S.D.S.); catalin.constantin@inflpr.ro (C.C.); 2 FOTOPLASMAT Department, National Institute for Laser, Plasma and Radiation Physics, 409 Atomistilor Street, 077125 Magurele, Romania; anca.bonciu@inflpr.ro; 3 Laser Section, National Institute for Laser, Plasma and Radiation Physics, 409 Atomistilor Street, 077125 Magurele, Romania; bercea.adrian@inflpr.ro; 4 Faculty of Physics, University of Bucharest, 405 Atomistilor Street, 077125 Magurele, Romania

**Keywords:** tungsten surfaces, crystallinity, nanostructured surface, plasma–tungsten surface interaction, hydrogen, deuterium, and helium plasmas, blisters, dust

## Abstract

In the current study, bulk tungsten material surfaces are exposed to hydrogen, deuterium, and helium plasmas in the radiofrequency domain (13.56 MHz) at an input power of 250 W using the hollow-cathode configuration. The ejected material is collected on titanium substrates at various distances (from 6 mm up to 40 mm). Therefore, the exposed tungsten materials are investigated for surface changes (blister occurrence, dust formation, or nano-structuration), along with the crystallinity, depending on the plasma’s exposure times (from 30 min up to 120 min for each plasma type). Also, the collected materials are analyzed (morphological, structural, and statistical investigations) for dust and dust film-like appearance. Plasma discharges are analyzed using two methods: optical emission spectroscopy, and single Langmuir probes, to emphasize the nature of the used plasmas (cold discharges, ~2 eV), along with the presence of tungsten emission (e.g., WI 406.31 nm, WI 421.31 nm) during the plasma lifetime. By using a dedicated protocol, a method was established for obtaining fusion-relevant tungsten surfaces in the hydrogen and deuterium plasma discharges. By using the implemented method, the current paper introduces the possibility of obtaining a new tungsten morphology, i.e., the dandelion-like shape, by using helium plasma, in which the W_18_O_49_ compound can be found.

## 1. Introduction

Tungsten (W) represents a significant component of the inner walls of facilities designed specifically for fusion, such as ITER, WEST, and DEMO. Due to its unique properties, such as low sputtering yields, a high melting point, low reactivity, etc., tungsten was anticipated to be a good material for the divertor area, providing additional stability to the lifetime of the thermonuclear plasma. As a result of particular instabilities, the plasma will interact with the inner vacuum vessel walls and encourage the production of other structures, such as dust, fuzz, etc. These structures can be redeposited on the divertor’s surface, leading to significant contamination of the divertor region or other PFCs (*plasma facing components*) [1,2,3,4,5]. Furthermore, dust may retain a sizable amount of tritium gas depending on its shape. The operation of the tokamak can also present a number of risks due to the presence of tungsten dust. One of the issues is the potential for a LOVA (*loss of vacuum accident*), in which contaminated (tritiated) tungsten dust can escape the vessel, despite safety precautions. This fact can lead to biological and environmental dangers, etc. [6,7,8]. In order to avoid critical operations/manipulation situations, in regards to both experimental data protection and experimental development, the need for computational approaches is in high demand, from cloud computing, to cybersecurity protection implementation of the fusion infrastructures, to computer simulation of the fusion phenomena. Here, the physical phenomena are predicted, in large part, thanks to the field of computer science. Calculations and experimental studies have revealed that dust material exhibits an essential tritium gas retention/release behavior. Many studies have been conducted on the behavior of hydrogen isotopes in tungsten, including the diffusion, permeation, and transport of hydrogen, deuterium, and tritium in the material, as well as the trapping of hydrogen, deuterium, and tritium by defects and other irradiation damage, all of which are thought to result in the formation of hydrogen bubbles that are responsible for the deterioration of the metal’s mechanical properties, leading to surface blistering [9,10,11,12,13]. Some theories regarding bubble generation mechanisms have been suggested in the literature, using a range of computational simulation approaches, such as plastic deformation, dislocation loop punching, and the aggregation of hydrogen vacancy complexes. Thus, dust morphology may act as an accelerator for gas retention. Additionally, as described for many samples, the morphology and/or structure of the dust particles would likely be affected by the interaction of dust with plasma. Furthermore, because of safety requirements, dust accumulation inside fusion plants poses a significant problem. Due to their ability to trap a significant amount of tritium during the plasma cycles, various investigations on the fusion-related gas retention of tungsten dust (such as tritium gas) focusing on this material [14,15,16]. The key parameters of safe manipulation, such as hydrogen retention in plasma-facing components (monoblocs); tritium adsorption/desorption; hydrogen, deuterium, and tritium solubility; diffusion and permeation in tungsten; or the evolution of deuterium bubbles in tungsten material, may be observed through experimental simulations [17,18,19,20,21,22]. Therefore, the monobloc surfaces that are exposed to plasma receive additional attention. Depending on how they were arranged, the surface monoblocs in this instance suffered damage at various levels. Important research involves deuterium transport, for instance, in QUEST, where hydrogen is taken into account for studies on hydrogen retention in tungsten, or in ITER facility-graded tungsten. As a result, a strong particle flux is used to establish the plasma contact at the divertor level (where the tungsten monoblocs are positioned). The most promising candidate to reduce the divertor heat load power is a tungsten divertor combined with a detached plasma. Maintaining a detached plasma is important because it helps to protect the walls of the tokamak from excessive heat and particle bombardment, which can damage the reactor’s components. Detachment causes the plasma temperature to drop, which lowers the heat load caused by ion and electron conduction and convection. Decreased ion particle flux is also crucial for lowering the load caused by surface recombination. Additionally, a detached plasma can have improved energy confinement properties, making it more efficient for sustaining fusion reactions. Divertor detachment involves low-temperature plasma at the plates, and it is frequently achieved in tokamaks currently in use by injecting fuel particles or impurities to increase the divertor power dissipation. The electron pressure computed using Langmuir probe measurements shows an approximately 90% loss near the strike point compared to the levels before impurity seeding, indicating a substantial pressure detachment. The electron temperature Te < 5 eV is highly desirable to prevent erosion in reactor feed devices [23,24,25,26,27]. It is important to note the lab-scale experiments regarding diffusivity inside bulk materials, i.e., in the case of deuterium, given the nature of the gases employed in the proposed fusion reaction (deuterium and tritium). In this case, the material results obtained by the proposed lab-scale plasma approach are relevant in the view of the detached plasma situation. For both cases, the temperature of the electrons is below 5 eV. A difference between them is related to the differences in densities, meaning that in the case of the detached plasma, the densities are in the domain of 10^16^–10^21^ m^−3^, whereas in our case, they are in the range of 10^16^–10^18^ m^−3^. Therefore, we have exposed the fact that the obtained tungsten surfaces emphasize the starting point of the possible surface damage, due to the plasma interaction phenomena. By using lab-scale approaches, the involved phenomena can be studied in safe steps.

In this line of the current study, we established a lab-scale method for obtaining fusion-relevant tungsten surfaces using hydrogen (H_2_), deuterium (D_2_), and helium (He) plasma discharges in a hollow-cathode configuration. Therefore, the exposed tungsten surfaces were analyzed for morphological changes using a scanning electronic microscope (SEM), and the crystallinity timeline of the materials was measured using the X-ray diffraction (XRD) technique. Moreover, for each plasma type discharge (hydrogen, deuterium, and helium), the ejected tungsten materials were collected on titanium substrates (Ti) at different distances from the cathode: 6 mm, 10 mm, 20 mm, and 40 mm. The collectors were analyzed, along the tungsten surfaces, using the same investigation methods. In addition, a preliminary statistical description was provided for the collected nanostructures. Furthermore, the plasma discharge was investigated using two methods: optical emission spectroscopy, and the single probe Langmuir method. The current study represents a continuation of our previous experiments [28,29] in order to achieve a method for stable, fast, and low-cost experiments which are suitable for obtaining fusion-relevant materials. The paper is divided into five sections: the *Introduction* (comprising the state of the art of the discussed subject), *Materials and Methods* (in which details regarding the procedure, the materials used, and methods are included), *Experimental Results* (showing the obtained results), *Discussion* (in which the occurring phenomena are debated), and *Conclusions* (in which the conclusions of the current study and suggestions for future experiments are expressed).

## 2. Materials and Methods

**Figure 1:**
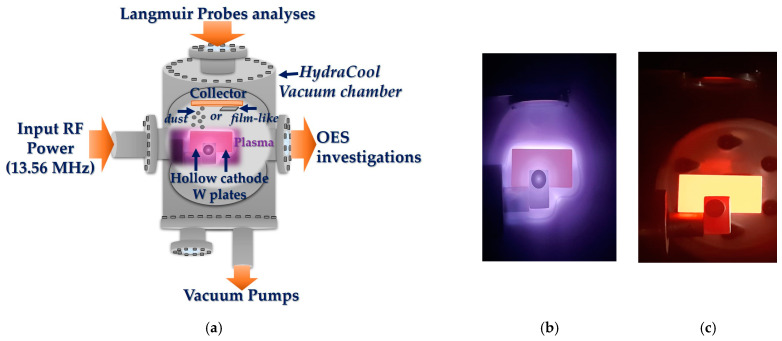
Plasma system design: (**a**) experimental hollow-cathode system design; (**b**) hot tungsten plates during D_2_ plasma ON; (**c**) hot tungsten plates after the plasma is switched off. The plasma images were obtained by using the Samsung S21 5G Ultra camera, which includes a system of cameras with the following technical configurations: 108 MP, f/1.8, 24 mm (wide), 1/1.33″, 0.8 µm, PDAF, Laser AF, OIS; 10 MP, f/4.9, 240 mm (periscope telephoto), 1/3.24″, 1.22 µm, dual pixel PDAF, OIS, 10× optical zoom; the third camera; 10 MP, f/2.4, 72 mm (telephoto), 1/3.24″, 1.22 µm, dual pixel PDAF, OIS, 3× optical zoom; 12 MP, f/2.2, 13 mm (ultrawide), 1/2.55″, 1.4 µm, dual pixel PDAF, Super Steady video.

The experimental system consisted of a hollow-cathode (HC) assembly [29] with a dust collector attached, which was placed inside a HydraCool vacuum chamber (water-cooled). The HC assembly was composed of two parallel tungsten plates (with a purity of 99.97%, PLANSEE supplier) with a 3 mm distance between them (Figure 1a).

Inside the deposition chamber, the tungsten plates are placed on the plasma source under dedicated support, which does not allow the manipulator to place them in the same position every time. The system setup was designed specially to keep the 4 mm thick plates in the same position; in this manner, we were able to compare the past results with the present findings. Each tungsten plate had a length of 30 mm, a width of 15 mm, and a thickness of 4 mm (Figure 1b,c) and was polished to a mirror-like state before the experiments [28]. The dust collector (from titanium) material, with a purity of 99.9% (NEYCO, Vanves, France), was placed above the HC assembly (see Figure 1a) at the following distances: 6 mm, 10 mm, 20 mm, and 40 mm. The tungsten plates were exposed to plasma for 30 min, 60 min, 90 min, and 120 min. The dust/film-like structures were collected for 30 min for each distance. In the experiments, we used a mass flow rate of 300 sccm: hydrogen gas (with a purity of 99.8% H_2_, SIAD, Bucharest, Romania), deuterium (99.8% D_2_, SIAD, Bucharest, Romania), and helium (99.9999% He, SIAD, Bucharest, Romania). The working pressure of 2 × 10^3^ Pa was necessary for achieving the plasma hollow-cathode phenomenon. We used the plasma discharge with an input power of 250 W in the radiofrequency domain (13.56 MHz) for all our experiments. The temperature reached by the tungsten plates was between 1000 °C and 1500 °C, which was measured using an optical pyrometer (OPTRIS Ctlaser, version 3MH CF3).

The surface morphology and chemical composition of the samples were investigated using scanning electron microscopy (SEM) and energy-dispersive spectroscopy (EDS). These analyses are conducted using an Apreo FEG High-Resolution Scanning Electron Microscope (HR-SEM), specifically the S LoVac model from Thermo Fisher Scientific Inc. in Hillsboro, OR, USA. This microscope is equipped with a Trinity detector system and is coupled with an EDAX Trident (EDS-EBSD-WDS) Analysis System from AMETEK Inc. in Mahwah, NJ, USA. The Apreo S LoVac HR-SEM is operated in high-vacuum mode, with an electron beam voltage of 10 kV.

The sample crystalline structure was investigated using X-ray diffraction (XRD). The XRD experimental setup consisted of a PANalytical X’Pert MPD diffractometer using Bragg–Brentano geometry. The XRD measurements are performed in the 30–90 2theta range with a step size of 0.02 and a 15 s acquisition time per step. The data processing was carried out using HighScore Plus software v5.1.

The spectral acquisition was obtained using a collimating lens that was attached to the optical fiber. This experimental setup arrangement allowed the electromagnetic radiation to be collected exclusively from the inter-cathode plates space (3 mm). The optical fiber was connected to a 1 m focal length monochromator (Yvon Jobin) equipped with 2400 grooves/mm diffraction grating. The plasma optical emission was collected through an external slit of 60 μm and recorded on a CCD detector (Andor iDus 420), placed at the exit slit. The CCD detector had dimensions of 1024 × 255 pixels, with each pixel measuring 26 × 26 μm. These particular parameters resulted in an instrumental profile at the half-width of approximately 0.02 nm.

Among the contact methods for plasma diagnostics, electrical probes are the least expensive, yet the fastest and most reliable diagnostic tools, providing values for plasma parameters such as plasma potential (*Vp)*, electron temperature (*Te)*, electron (*Ne),* and ion (*Ni)* densities, as well as the distribution functions (EEDF) of the charged particles (electrons and ions) [30,31,32]. A complete fixed probe system consists of an interface unit (EPIU—ESPION Probe Interface Unit), a gas-cooled, radio-frequency electrostatically compensated probe, and connection cables. The probe also includes a reference electrode for use in high-impedance plasmas. A standard system uses the probe current in the range of [1 mA–1 A], at a peak voltage in the range of [−200–100 V].

## 3. Experimental Results

Hydrogen, deuterium, and helium plasma discharge experiments were conducted by using the experimental parameters presented in Table 1. The goal was to highlight the changes that occurred at the bulk tungsten surface material in a “soft” manner by using a cold plasma approach under safe conditions. For each plasma type, a timeline of the occurring structures was established, at the same time obtaining an experimental calibration.

### 3.1. Tungsten Plate Morphology after Hydrogen, Deuterium, and Helium Plasma Exposure

**Figure 2:**
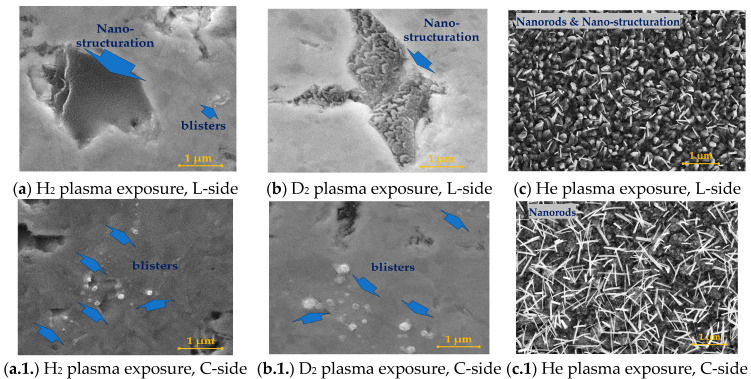

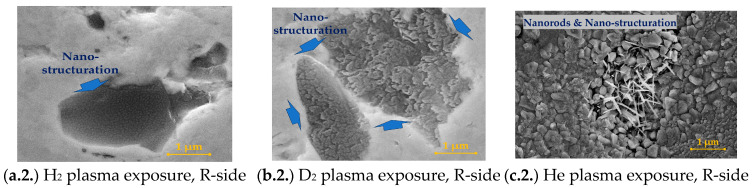
SEM images of the tungsten surface plates after hydrogen, deuterium, and helium plasma exposure for 30 min, including the influence of the nature of the gas.

Tungsten plates were analyzed using the SEM method in order to emphasize the influence of the nature of the gas used in the plasma discharges. In Figure 2, the SEM images of the tungsten surface plates can be observed after 30 min of exposure to hydrogen, deuterium, and helium plasma. For each tungsten plate, we took three regions for investigation: *L-side* is the left-hand region; *C-side* is the center region, and *R-side* is the right-hand region. Compared to the tungsten plate, each side is considered to have an area of around 7 mm × 9 mm, and its location on the plate can be seen in the reference [28].

**Figure 3:**
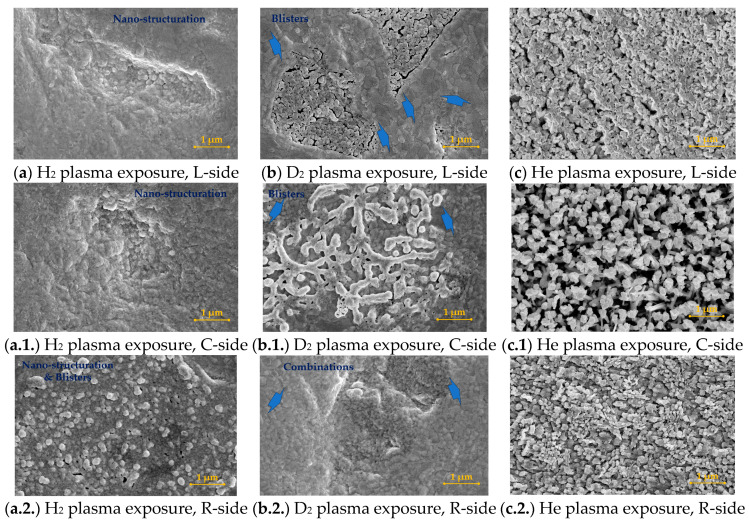
SEM images of the tungsten surface plates after hydrogen, deuterium, and helium plasma exposure for 120 min, including the influence of the nature of the gas.

In Figure 3, the SEM images of the same tungsten plates are emphasized, but this time, after 120 min of exposure to the hydrogen, deuterium, and helium plasma discharges. Therefore, the significance of each gas used can be established; while hydrogen and deuterium are well-known for their high diffusivity property, helium is a perfect candidate gas for creating new-like vertical structures because of the helium bubble migrations inside the tungsten bulk material. The scientific explanation of the phenomena that occurred in our study is provided in Section 4.

**Figure 4:**
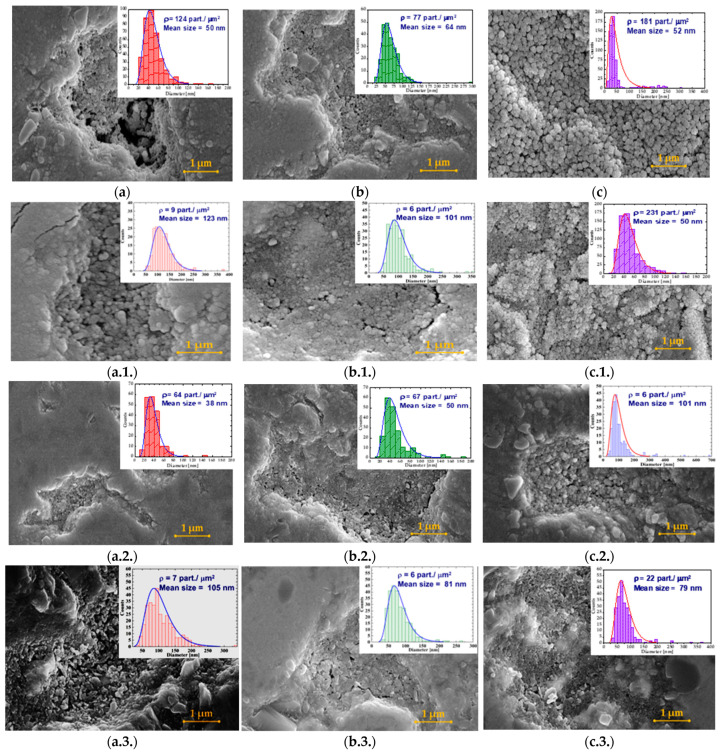
SEM images of the materials deposited on the Ti collectors after hydrogen, deuterium, and helium plasma exposure, at distances 6–40 mm. The influence of the nature of the gas is as follows: (**a**) 6 mm, after H_2_ plasma exposure; (**a.1.**) 10 mm, after H_2_ plasma exposure; (**a.2.**) 20 mm, after H_2_ plasma exposure; (**a.3.**) 40 mm, after H_2_ plasma exposure; (**b**) 6 mm, after D_2_ plasma exposure; (**b.1.**) 10 mm, after D_2_ plasma exposure; (**b.2.**) 20 mm, after D_2_ plasma exposure; (**b.3.**) 40 mm, after D_2_ plasma exposure; (**c**) 6 mm, after He plasma exposure; (**c.1.**) 10 mm, after He plasma exposure; (**c.2.**) 20 mm, after He plasma exposure; and (**c.3.**) 40 mm, after He plasma exposure. A statistical description was performed and attached for each sample.

Along with W plate, the exposure to the plasma discharges were recorded by vertical collectors, placed at various distances, in order to catch the ejected materials. This design was inspired by the fusion-relevant scenario, in which the plasma interacts with the W divertor region, ejecting materials at various distances. Figure 4 shows the evolution of the W material collected for each plasma discharge. From the top-view SEM images, a tendency to form film-like dust structures from merged dust structures can be observed. An interesting result is obtained from the use of helium plasma discharge. In this case, the dust arrangement in the film can be clearly seen in Figure 4c,c.1.,c.2. Statistics were collected from dedicated SEM images in order to emphasize the dimensions of the dust from the film-like structures. The dust dimensions were obtained in manual mode using ImageJ software. Taking into account the gas diffusivity property and the small non-uniform discharge, we gathered only rudimentary statistics to emphasize the nature of the collected materials and their statistical range. In the attached data, it can be seen that the mean dust size is around 50 nm. It is worth mentioning that the measured dust occurred in a cauliflower shape, meaning that the W dust is shown in much smaller dimensions. Future measurements are foreseen in terms of TEM measurements in order to establish the dust dimension.

#### Timeline of Tungsten Plates Surface Morphologies under He Plasma Exposures

**Figure 5:**
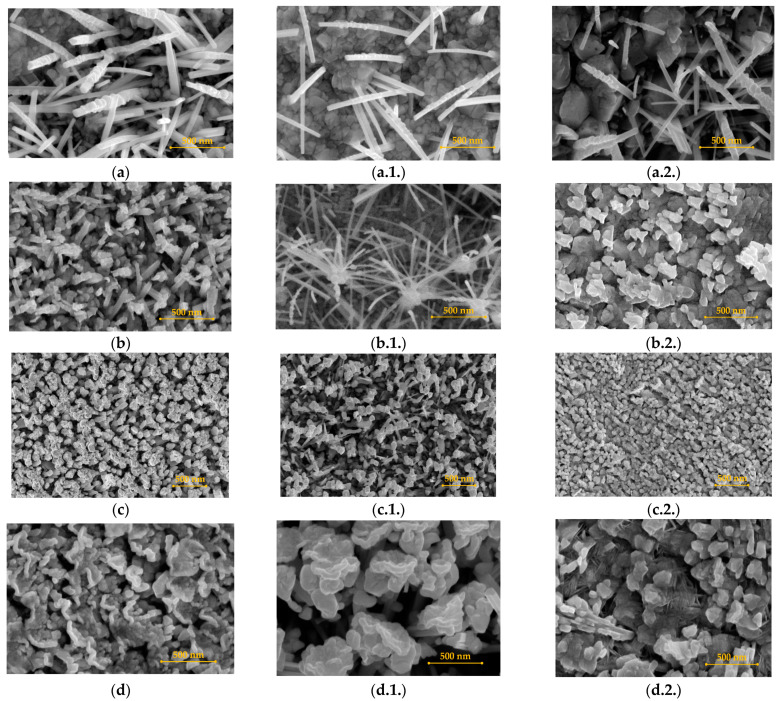
SEM images of the tungsten surface plates after helium plasma exposure. The influence of the time exposure: (**a**) after 30 min, left-hand side (L-side); (**a.1.**) after 30 min, center side (C-side); (**a.2.**) after 30 min, right-hand side (R-side); (**b**) after 60 min, left-hand side (L-side); (**b.1.**) after 60 min, center side (C-side); (**b.2.**) after 60 min, right-hand side (R-side); (**c**) after 90 min, left-hand side (L-side); (**c.1.**) after 90 min, center side (C-side); (**c.2.**) after 90 min, right-hand side (R-side); (**d**) after 120 min, left-hand side (L-side); (**d.1.**) after 120 min, center side (C-side); and (**d.2.**) after 120 min, right-hand side (R-side).

In the current section, an interesting process of helium plasma treatment of W bulk surfaces is presented. In this case, in Figure 5, we present the timeline of the surface changes for the same three measured areas: L-side, C-side, and R-side.

Therefore, the influence of the plasma treatments can be distinguished: the margin regions (L-side and R-side) seem to exhibit the same trend, whereas in the center part, the changes are more intense and distinct away from the plate margins. In the central part, the occurrences of W stake morphology (Figure 5a.1.) can be seen, evolving into a dandelion-like morphology after 60 min of plasma exposure (Figure 5b.1.). By continuing the plasma exposure in steps (30 min for each step), it can be seen that the dandelion-like structures continue their transformation into very thick W flakes.

### 3.2. Tungsten Bulk and Dust XRD Investigation after Hydrogen, Deuterium, and Helium Plasmas Exposure

**Figure 6:**
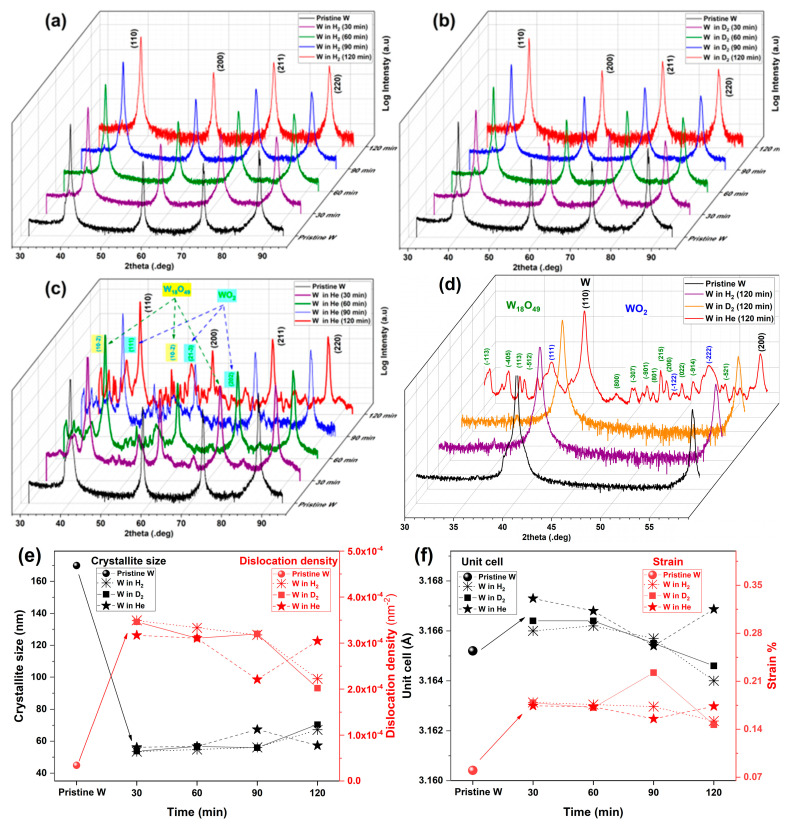
**XRD analyses for W plates:** (**a**) XRD diffraction patterns of W in H_2_ plasma vs. time; (**b**) XRD diffraction patterns of W in D_2_ plasma vs. time; (**c**) XRD diffraction patterns of W in He plasma vs. time; (**d**) comparison of XRD diffraction patterns of W in H_2_, D_2_, and He plasma at 120 min; (**e**) crystallite size and dislocation density of cubic W vs. expositor time; (**f**) unit cell volume and strain of cubic W vs. expositor time.

The diffraction patterns of the samples are presented in Figure 6, and the corresponding measurement data are inserted in Table 2. The pristine W (before plasma treatments) shows a cubic phase of W (JCPDS file: 004-0806), with a polycrystalline nature, as evidenced by the presence of the (110), (200), (211), and (220) lattice planes (Figure 1a).

When W is exposed to the H_2_ and D_2_ plasma, the crystallographic structure of the material remains unchanged, and no additional phases are detected, as shown in Figure 6a,b. This is not the case for the W exposure to the He plasma (Figure 6c). In He plasma, W undergoes crystallographic changes characterized by the presence of new additional peaks in the sample diffractogram. These peaks reveal the existence of WO_2_ (JCPDS file: 008-4852) and W_18_O_49_ (JCPDS file: 005-4539) in the samples, but the cubic W crystal structure remains the main constituent. Here, this phenomenon is unique to W exposure to He plasma and is not seen for the H_2_ and D_2_ plasmas, as shown in Figure 6d.

For the cubic W present in all samples, the medium crystallite diameter has been calculated via the Scherrer equation. During W treatment in the H_2_, D_2_, and He plasmas, the crystallite size decreases from the initial 179 nm to 67, 70, and 57 nm, respectively, for the three gases after 120 min of exposure time (see Figure 6e). The decrease in crystallite size is quite important, and it is coupled with an increase of one order of magnitude in the dislocation density (3.4 × 10^−5^ nm^−2^ vs. 2.2 × 10^−4^ nm^−2^ for 120 min in H_2_).

The evolution of the unit cell and strain of cubic W, as a function of gas nature and exposure time, shows an interesting tendency (see Figure 6f). In the first 30 min of plasma exposure, the W unit cell increases in size. Then, as the exposure time reaches 60/90 min, the unit cell decreases, and at 120 min, the three gases show their different effects. The same characteristics are seen in the strain evolution of W. As the expositor time increases the strain goes from 0.08% in pristine W to 0.152% in H_2_, 0.146 D_2_, and 0.173% in He (after 120 min). These crystallographic changes in W in terms of crystallite size, dislocation density, unit cell size, and strain show that from a crystallographic point of view, the nature of the gas used in this process is significant. Regarding the comparison of the gas nature, H_2_ and D_2_ have a similar effect on W crystallography, while He shows the highest influence.

**Figure 7:**
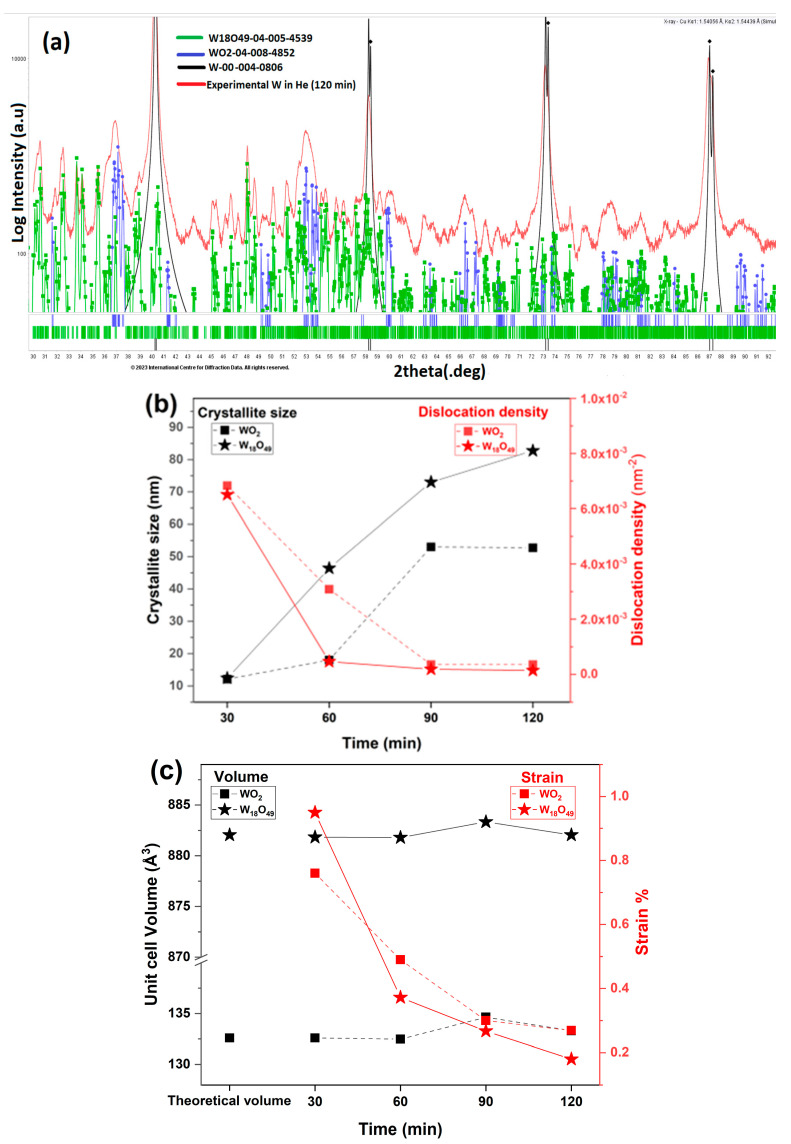
**XRD analyses for W plates:** (**a**) the XRD diffraction patterns of experimental and computed data using the PDF-4+ Database (ICDD PDF-4+ 2012 & PDF-4 Organics 2014 Databases. International Center for Diffraction Data, Newtown Square, PA, USA (2014)) of W in He plasma; (**b**) crystallite size and dislocation density of WO_2_ and W_18_O_49_ vs. He plasma expositor time; (**c**) unit cell volume and strain of WO_2_ and W_18_O_49_ vs. He plasma expositor time.

As mentioned before, when W is exposed to He, peaks of WO_2_ and W_18_O_49_ are seen in the XRD measurements. Figure 7a (computed using PDF-4+ Database) shows that all the peaks are fully resolved by WO_2_, W_18_O_49_, and the cubic W. WO_2_ and W_18_O_49_ both exhibit a monoclinic structure with the P2/m space group. WO_2_ and W_18_O_49_ are defined by the presence of three unequal crystallographic axes, with one of them perpendicular to a unique plane, leading to the unique angles between the crystallographic axes, making it distinct from cubic W. By evaluation using the Scherrer equation, the crystallite size of WO_2_ and W_18_O_49_ are shown in Figure 7b. Initially, after 30 min of expositor time, the crystallites are small (12.4 nm for W_18_O_49_ and 12 nm for WO_2_). But, as the expositor time increases, the crystallites grow and reach 82 nm for W_18_O_49_ and 52 nm for WO_2_ (at 120 min).

The crystallite size of WO_2_ reaches a plateau after 90 min (around 50 nm). W_18_O_49_ is known to have an acicular morphology [33,34], and its crystallite growth can explain the presence of the structures seen in the SEM images.

In W_18_O_49_, the crystallite grew by 583.33% (30 min vs. 120 min) during the whole process. Also, the dislocation density dropped by one order of magnitude (from 6.8 × 10^−3^ to 3.5 × 10^−4^ for WO_2_ and 6.5 × 10^−3^ to 1.4 × 10^−4^ for W_18_O_49_) after 30 min vs. 120 min of expositor time. As seen in Figure 7c, the computed unit cell volumes of WO_2_ and W_18_O_49_ are close to their theoretical values. The strain in both WO_2_ and W_18_O_49_ decreases by an order of magnitude with increases in W He plasma expositor time.

**Figure 8:**
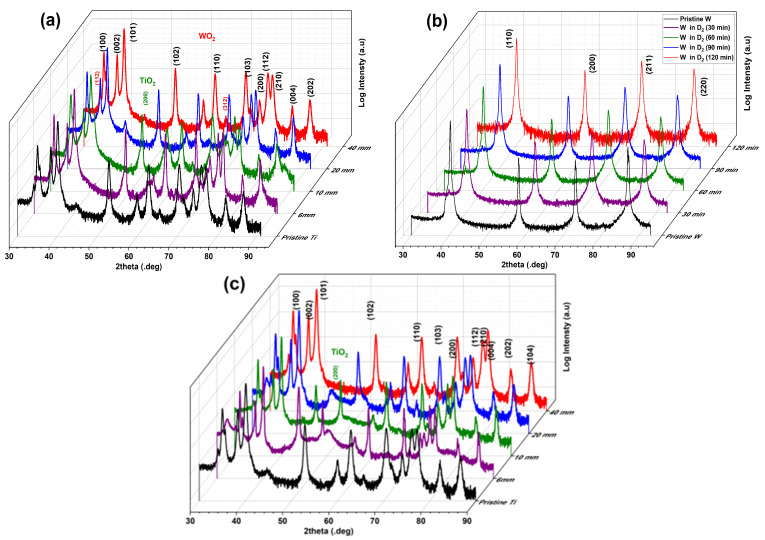
**XRD analyses for the collected materials:** (**a**) XRD diffraction patterns of W collected on Ti in H_2_ plasma vs. time; (**b**) XRD diffraction patterns of W collected on Ti in D_2_ plasma vs. time; (**c**) XRD diffraction patterns of W collected on Ti in He plasma vs. time.

A shown in Figure 8, for the collected W exposed to H_2_, D_2_, and He plasma and collected on Ti substrates, there are no observable crystalline metallic cubic W peaks. Besides Ti peaks, there are very small traces of TiO_2_ and WO_2_, but these are not sufficient to clearly distinguish between the samples.

### 3.3. OES Analyses of the Hydrogen, Deuterium, and Helium Plasmas Exposoures

**Figure 9:**
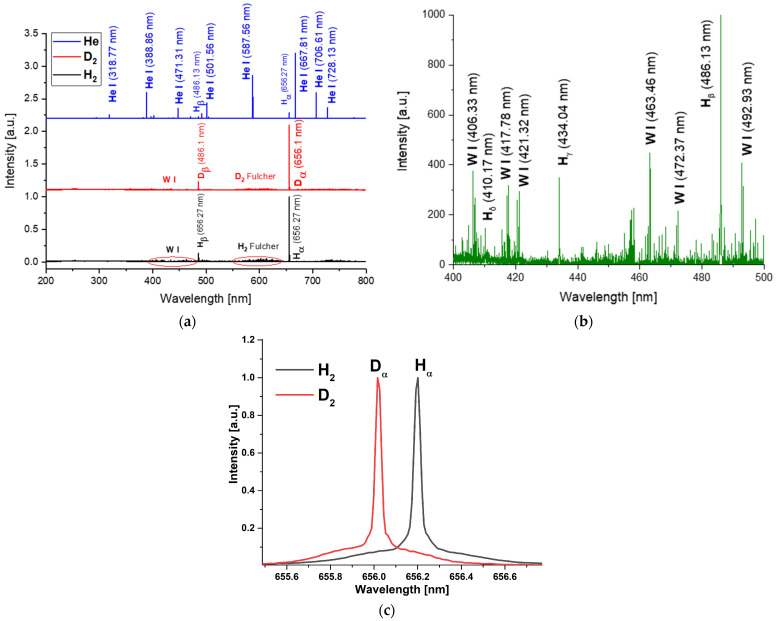
**OES measurements of H_2_, D_2_, and He plasmas:** (**a**) general spectra acquired for H_2_, D_2_, and He; (**b**) examples of W emission lines observed; (**c**) Hα and Dα line shapes recorded in hydrogen and deuterium plasmas, respectively.

The experiments were carried out using the same experimental setup, changing only the working gas: H_2_, D_2_, and He. The general spectra for these experiments are presented in Figure 9a, and the corresponding emission lines are inserted in Table 3. For hydrogen and deuterium, the spectra are quite similar. There are two distinct zones: one between 400–500 nm and another between 550–650 nm. In both cases, emissions from excited tungsten lines can be observed in the first zone (Figure 9b). The second zone is mainly occupied by the Fulcher bands of hydrogen and deuterium, when their respective gases are used. The Balmer series is also visible for both gases up to Hδ and Dδ, respectively (Figure 9b).

On the other hand, when using He, the main emission comes from excited He. The excited tungsten lines are much weaker and fewer compare with those of hydrogen and deuterium. Hα and Hβ can be still observed, suggesting that due to diffusion phenomena, the hydrogen is removed from the chamber walls.

In Figure 9c, the line shapes of the hydrogen and deuterium lines observed when the working gases are H_2_ or D_2_ are presented. Similar line shapes of the Balmer series are presented in several studies in the hallow-cathode regime [35,36], for which the authors explain and analyze the presence of these profiles. The authors conclude that two distinct regions can be observed: a central narrow peak and a plateau made up of two wings—one shifted to blue and one to red. The central area broadening is a result induced mainly by electrons (Stark broadening). The plateau, on the other hand, is characterized by two wings, one extending toward the blue side and the other toward the red side of the spectrum. This plateau is a result of the optical emission from rapidly excited atoms moving towards and away from the detector (Doppler broadening). In the case of the plasma generated in He, the plateau is not observed.

### 3.4. Single Langmuir Probe Measurements of the Hydrogen, Deuterium, or Helium Plasmas Exposures

**Figure 10:**
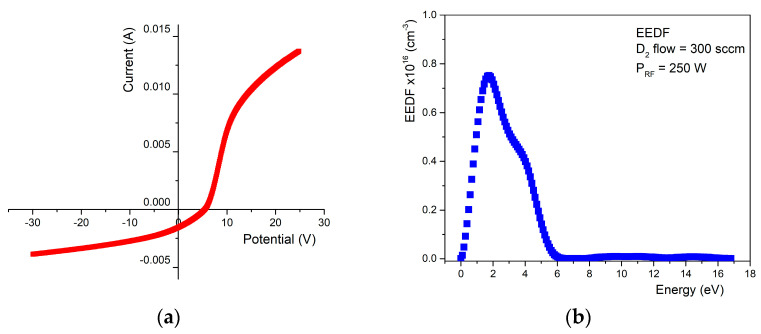
**Single Langmuir probe measurements:** (**a**) current-voltage characteristic; (**b**) electron energy distribution function measured, in the case of deuterium usage.

The tip of the probe is made of a material resistant to high temperatures, usually a tungsten or platinum rod or wire with a diameter of 0.1–1 mm. The rod is inserted into a thin ceramic tube, usually made of alumina, to be isolated from the plasma, except for contact with a short portion of the exposed tip, approximately 2–10 mm long. For this experiment, we used three different gases, i.e., hydrogen, deuterium, and helium. The electrical probe measurements took place in the channel created by the two electrodes of the plasma source. Figure 10 presents examples of the I-V characteristics and the electron energy distribution function measured, in the case of deuterium plasma.

The electrical measurements were performed for each plasma discharge type in time, so the presented values are, in fact, the mean value of these measurements. The average values of the most important plasma parameters, generated in different gases, are presented in Table 4.

## 4. Discussion of the Experimental Results

The current studies are focused on achieving a suitable method for obtaining fusion-relevant materials using a low-cost, safe, and environment-friendly experimental approach, in this case, by combining the phenomena of the used gases (e.g., diffusion, gas bubble migration, deformation of the bulk matrix) with the cold plasma environment (~2 eV from the electrical measurements) and in the presence of high temperatures (around 1500 °C), obtaining tungsten surfaces with a high area by soft processing. This approach was employed for an extreme case of a tungsten divertor in the plasma detached mode, which was exposed to the plasma over several time periods, without changing the damaged monoblocks. The proposed experimental method was developed, taking into account the lab system geometry constraints, along with the experimental research capacity, offering relevant results in regards to the commencement of tungsten damage. Herein, the methods consist of using mirror-like polished tungsten plates, with dimensions of 30 mm × 15 mm × 4 mm, placed in a parallel arrangement, with a distance of 3 mm between them. Tungsten plates are placed inside a cooled vacuum chamber to remove the impurities by reaching an inner pressure of 1 Pa. Subsequently, a continuous gas flow rate of 300 sccm is pumped inside, increasing the internal pressure up to a value of 2 × 10^3^ Pa. This pressure is necessary in order to confine the plasma discharge between the parallel tungsten plates. A time period of 30 min of plasma exposure, followed by a pause of 45 min in the vacuum atmosphere, offers the necessary experimental control for further manipulation of the tungsten plates, namely material investigations. Using this method highlighted the fact that by using hydrogen or deuterium plasma discharge, the crystallinity of the bulk material is maintained in time, i.e., a 30 min plasma exposure is equivalent to a 120 min exposure, while the surface of the plates shows an increase in the blister zones with increasing time exposures. This method, when applied to helium plasma, offers a control of the desired surface morphologies. Herein it was shown that by using different time exposures, we can obtain dedicated tungsten surface morphologies.

### 4.1. The Influence of the Hydrogen Plasma on Tungsten Bulk Materials

In the case of using hydrogen plasma, the bulk material was exposed, in steps, for a total time of 120 min. Each step consists of a plasma exposure for 30 min, followed by a cooling time in a vacuum for about 45 min. By cooling down the tungsten plates in a vacuum, we prevent the formation of a higher degree of oxidation on the material’s surface. In addition, the bulk material was analyzed after each plasma treatment in order to establish a timeline of the changes induced by the plasma.

Figure 2 shows the SEM images for the W bulk after the first step of plasma exposure. The first proof of the plasma exposure is the appearance of blisters (Figure 2a.1.) and the nano-structuration in the margin sides of the bulk materials (Figure 2a,a.2.). As we have shown in our previous studies, the diffusion phenomenon is present in the case of hydrogen gas usage. Therefore, as we previously shown, hydrogen has a high degree of diffusivity inside materials, inducing the plastic deformation of the material matrix, resulting in hydrogen bubbles [37] due to material matrix vacancies. Depending upon on vacancy degree, the hydrogen accumulation inside the matrix can vary. However, the tungsten bulk materials used in this study were purchased from the same supplier, meaning that the bulk material should be considered equivalent, in terms of vacancies, for all of our conducted studies. Subsequently, the appearance of the nano-structuration in the first 30 min of the plasma exposure is due to the input power increase, emphasizing the possibility of higher amounts of hydrogen accumulation on the tungsten surface than those noted in our previous studies.

Figure 3 emphasizes the results of tungsten bulk after 120 min of exposure to the plasma. As can be observed in all images, there are slightly different degrees of nano-structuration and blistering occurrence. From our previous studies, it seems that the hydrogen plasma is not homogenous between the tungsten plates (with a length of 30 mm), exhibiting different degrees of blistering. The result was expected because we are using a cold plasma, which by its nature, forms blisters from the energetic ions combined with the un-ionized gas, leading to a different depth profile of the gas bubbles inside the matrix of the bulk material, along with various blisters repartitioning on the exposed surface.

An interesting result is related to the materials collected at different distances from the hollow cathode geometry (6 mm, 10 mm, 20 mm, and 40 mm). Figure 4 shows the SEM images of the collected material. Compared to the results of our previous studies, in this study, we have obtained film-like dust. Statistics obtained from two dedicated SEM images highlight the fact that the mean size of the dust-like structure is in the range of 38–50 nm. Moreover, the manually measured dust exhibits a cauliflower-like morphology, which means that the obtained particles are much smaller. Future dust morphological investigations (TEM) are planned in the near future for the collection of more scientific details.

From the point of view of crystallinity, the exposed tungsten bulk remains stable in time, meaning that we have achieved a stable experimental plateau. However, tungsten emission lines can be observed (see Table 3) during plasma lifetimes, meaning that during plasma interaction with bulk tungsten, metallic tungsten vapors, present inside the plasma, are formed, leading to the formation of small dust-like structures and dust film-like structures. From our previous studies, we know that tungsten dust is formed inside the plasma and is transported to the collector.

From the electrical measurements, we have obtained a mean value of the electron temperature of about 1.73 eV, and electron and ion densities of 3.15 × 10^16^ m^−3^ and 1.52 × 10^18^ m^−3^, emphasizing the cold nature of the plasma. Therefore, part of the injected gas will remain un-ionized, contributing to the hydrogen diffusion because of the presence of the elevated temperature due to the direct power application on the bulk tungsten material.

Analyzing the results of the investigation methods, it can be said that the nanostructure present on the bulk tungsten surface is induced by the hydrogen diffusion on a large area, mainly on the bulk surface.

### 4.2. The Influence of the Deuteriumn Plasma on Tungsten Bulk Materials

Bulk tungsten material exposed to the deuterium plasma discharge underwent the same protocol used in the case of hydrogen plasma exposure, meaning a plasma exposure of 30 min, followed up by a colling process in the vacuum environment for 45 min. In addition, as can be observed from Figure 2b,b.1.,b.2., the nano-structuration (see Figure 2b,b.2.) and blistering (see Figure 2b.1.) occur after 30 min. By increasing the exposure time, blisters are formed, which are prominent in the central part of the exposure (see Figure 2b.1.).

As in the case of hydrogen, deuterium exhibits the same property of diffusion in the materials. Deuterium gas and the ionized deuterium diffuse inside bulk tungsten material, forming deuterium bubbles, which migrate towards the surface material and deform it. Depending on the deuterium gas loading inside the bulk, the bubbles continue to grow, forming blisters of various dimensions on the bulk tungsten surface [38,39].

The collected tungsten materials exhibit the same morphologies, dust, and dust film-like structures (see Figure 4b,b.1.–b.3.). Statistics obtained highlight a dust dimension in the range of 50–64 nm. There are no important changes from the point of view of crystallinity during 120 min of plasma exposure. As in the case of hydrogen plasma, it reached a plateau of material stability.

Tungsten emission lines are present in the deuterium plasma discharge, confirming the presence of the tungsten metallic vapor inside the plasma. Electrical measurements highlight an electronic temperature of 2.13 eV, as well as electron and ion densities of 2.42 × 10^16^ m^−3^ and 1.17 × 10^18^ m^−3^, respectively, confirming again the cold nature of the used plasma.

Compared with the bulk tungsten exposed to hydrogen plasma, in the case of deuterium plasma exposure, the blistering phenomena is more prominent in terms of gas retention. Increasing the occurrence of blisters on the material surface suggests that the gas bubbles trapped at different depth levels inside the bulk material have migrated toward the surface. A higher number of surface blisters means that deuterium is trapped in a higher amount near the surface, taking into account that each blister is filled with deuterium gas.

Future investigations are planned in order to obtain a depth profile regarding the deuterium penetration inside the bulk tungsten matrix.

### 4.3. The Influence of the Helium Plasma on Tungsten Bulk Materials

In the case of helium plasma exposure, the newly formed structures are very different compared to those formed by using hydrogen and deuterium plasmas. If, in the case of hydrogen and deuterium plasma exposure, the surface bulk structures consist of blistering and nano-structuration morphologies, then helium plasma represents an ideal approach for producing nano-rods, with fuzz-like and dandelion-like morphologies, and vertical structures, depending on the parameters used. We have used the same protocol for the plasma exposure steps as those used in the case of hydrogen and deuterium plasma exposures. In addition, after 30 min of exposure to the helium plasma, the tungsten surface is covered by nano-rod structures (see Figure 2c,c.1.,c.2.), with a prominent presence in the center of the exposed bulk material (see Figure 2c.1.). However, the SEM images emphasize two types of structures, including a significant nano-structuration, whereas the surface is clearly changed to a dust-like shape (see Figure 2c.2.), combined with the appearance of nano-rod morphologies (see Figure 2c.1.). It is worth mentioning that the nano-rods are stuck to the bulk material, meaning that the helium gas and plasma played an important role in the growth of these shapes. As in the case of hydrogen and deuterium, helium exhibits the possibility of producing nano-structures on the exposed surface due to gas diffusion, bubble migration, and bubble accumulation at the surface of the exposed material. Because the surface is exposed to the plasma discharge for a long period of time, this is equivalent to a high amount of gas bubble retention and migration, forming different vertical structures, depending upon plasma conditions. In the case of helium, it can be observed from the SEM images that the nano-rods are stuck in the bulk material, leading to the conclusion that the nano-rods grew because of the gas bubble retention and migration [40,41]. Thus, on the tungsten plate, we can identify slight shapes in the investigated regions. This is due to the fact that the plasma was not homogeneous on the surface, leading to different local temperatures, resulting in the formation of slightly different nano-structure shapes.

The most important results are highlighted in Figure 5, where the timeline of the surface changes in three regions during helium plasma exposure. Briefly, starting from a mirror-like polished tungsten surface, after 30 min, the surface suffers an important nano-structuration, along with the appearance of the nano-rod shapes. By continuing the plasma exposure, the nano-rods suffer a change in the dandelion-like shape (see Figure 5b.1.) in the center, along with the appearance of tungsten flakes in the margins. After another 60 min, the surface morphologies evolve into these flakes. It is worth mentioning that after each plasma exposure step, the morphologies evolve, creating a method for obtaining a precise structure. The changes in the morphologies are validated by XRD measurements, whereas the appearance of the oxygen in the new structures, W_18_O_49_, led to the formation of the new dandelion, fuzz-like shapes. Compared to the case of hydrogen and deuterium plasma, herein, the electron temperature is 2.89 eV, with electron and ion densities of 4.66 × 10^16^ m^−3^ and 1.78 × 10^18^ m^−3^, respectively, emphasize the cold nature of the used plasma. For all studies, the plasmas are very stable, revealing a useful approach for obtaining surface changes in tungsten in a soft manner.

## 5. Conclusions

The present study describes a stable and safe method for obtaining fusion-relevant tungsten surfaces by using a cold plasma (~2 eV) approach. The method consists of exposing the tungsten surfaces in well-determined time steps (30 min), at a working pressure of 2 × 10^3^ Pa, with an input power of 250 W, and by using the W plate geometries expressed in Section 2. In this case, hydrogen and deuterium plasmas were used in order to obtain tungsten surfaces with different blister occurrences. Deuterium plasma facilitates the appearance of a highly blistered surface, compared to that resulting from hydrogen plasma. Exposing the surfaces to the hydrogen and deuterium plasma will increase the blistering occurrences. In the case of helium plasma, the surface morphologies evolve, depending on the time step exposure. Moreover, structures like nano-rods, thick fuzz-like structures, dandelion-like formations, and nano-structuration are acknowledged. XRD measurements have shown a crystallographic stability of the tungsten material exposed to hydrogen and deuterium plasmas, whereas for the helium plasma exposures, the crystallinity of the material changes at each exposure step was in accordance with the SEM measurements. The electrical measurements have shown the cold nature of the used plasmas.

Future experiments are proposed in order to analyze the uniformity of the electronic temperature, along with tungsten bulk material properties, exposed to the hydrogen and deuterium plasmas.

## Figures and Tables

**Table 1:** Experimental parameters for hydrogen, deuterium, and helium plasma discharges.

Gas Name	Material Plates	Inlet Gas Pressure	Working Pressure	Input Power	Time Exposure
**Hydrogen (*H*_2_)**	Polished Tungsten (W)	300 sccm	2 × 10^3^ Pa	250 W	30 min
					60 min
					90 min
					120 min
**Deuterium** **(*D*** **_2_** **)**	Polished Tungsten (W)	300 sccm	2 × 10^3^ Pa	250 W	30 min
					60 min
					90 min
					120 min
**Helium** **(*He*)**	Polished Tungsten (W)	300 sccm	2 × 10^3^ Pa	250 W	30 min
					60 min
					90 min
					120 min

**Table 2:** The computed data from XRD measurements.

Gas	Material	Exposure Time (min)	Crystallite Size (nm)		Dislocation Density × 10^−4^ (nm^−2^)		Strain (%)	
			Value	Standard Deviation	Value	Standard Deviation	Value	Standard Deviation
-	Tungsten	-	170	±29.6	0.34	±0.12	0.080	±0.019
H_2_	Tungsten (W)	30	53.50	±12.29	3.49	±1.6	0.179	±0.034
		60	54.80	±12.8	3.32	±1.55	0.176	±0.033
		90	56.10	±12	3.17	±1.35	0.173	±0.033
		120	67.07	±17.55	2.22	±1.163	0.152	±0.021
D_2_	Tungsten (W)	30	53.77	±11.15	3.45	±1.43	0.178	±0.034
		60	56.75	±11.78	3.10	±1.28	0.171	±0.032
		90	55.92	±13.5	3.19	±1.54	0.222	±0.035
		120	70.37	±15.78	2.01	±0.905	0.146	±0.026
He	Tungsten (W)	30	56.17	±12.11	3.16	±1.36	0.174	±0.033
		60	56.75	±12.21	3.10	±1.33	0.172	±0.033
		90	67.30	±17.91	2.20	±1.17	0.155	±0.022
		120	57.30	±12.33	3.04	±1.31	0.173	±0.033
	WO_2_	30	12.1	±2.23	68	±25.17	0.76	±0.11
		60	18	±3.48	30	±11.93	0.49	±0.059
		90	53	±10.73	3.55	±1.44	0.3	±0.044
		120	52.73	±10.17	3.59	±1.38	0.269	±0.037
	W_18_O_49_	30	12.40	±2.56	65	±26.85	0.95	±0.210
		60	46.40	±9.82	4.64	±1.96	0.37	±0.045
		90	73.00	±18.71	1.87	±0.96	0.26	±0.036
		120	82.77	±19.11	1.45	±0.67	0.18	±0.034

**Table 3:** OES lines obtained in H_2_, D_2_, and He plasma discharges.

Optical Emission Spectroscopy Lines—Wavelength [nm]							
**W I**	**Hα**	**Hβ**	**H_2_** **Fulcher**	**Dα**	**Dβ**	**D_2_** **Fulcher**	**He I**
406.33	656.27	486.13	590–650	656.10	486.31	600–640	318.77
417.78							388.86
421.31							471.31
434.04							501.56
463.46							587.56
472.37							667.81
492.93							706.61
							728.13

**Table 4:** Electrical measurements values for H_2_, D_2_, and He plasma discharges.

Name	*Te* (eV)	*Ne* × 10^16^ (m^−3^)	*Ni* × 10^18^ (m^−3^)	*Vp* (V)
**Hydrogen** (***H*_2_**)	1.73 ± 0.09	3.15 ± 0.07	1.52 ± 0.014	10.75 ± 0.36
**Deuterium** (***D*_2_**)	2.13 ± 0.03	2.42 ± 0.13	1.17 ± 0.004	9.83 ± 0.013
**Helium** (***He***)	2.89 ± 0.13	4.66 ± 0.15	1.78 ± 0.07	17.94 ± 0.29

## Data Availability

The data are contained within the article.
